# Analysis of *O*-glycoforms of the IgA1 hinge region by sequential deglycosylation

**DOI:** 10.1038/s41598-020-57510-z

**Published:** 2020-01-20

**Authors:** Yukako Ohyama, Hisateru Yamaguchi, Kazuki Nakajima, Tomohiro Mizuno, Yukihiro Fukamachi, Yasuto Yokoi, Naotake Tsuboi, Daijo Inaguma, Midori Hasegawa, Matthew B. Renfrow, Jan Novak, Yukio Yuzawa, Kazuo Takahashi

**Affiliations:** 1 Department of Nephrology, Fujita Health University School of Medicine, Toyoake, Japan; 2 Institute for Comprehensive Medical Science, Fujita Health University, Toyoake, Japan; 3 Center for Research Promotion and Support, Fujita Health University, Toyoake, Japan; 4 Analytical Pharmacology, Faculty of Pharmacy, Meijo University, Nagoya, Japan; 5 Mitsui Knowledge Industry, Tokyo, Japan; 6 Departments of Biochemistry and Molecular Genetics and Microbiology, University of Alabama at Birmingham, Birmingham, AL USA; 7 Department of Biomedical Molecular Sciences, Fujita Health University School of Medicine, Toyoake, Japan

**Keywords:** Glycobiology, IgA nephropathy

## Abstract

A common renal disease, immunoglobulin A (IgA) nephropathy (IgAN), is associated with glomerular deposition of IgA1-containing immune complexes. IgA1 hinge region (HR) has up to six clustered *O*-glycans consisting of Ser/Thr-linked *N*-acetylgalactosamine with β1,3-linked galactose and variable sialylation. IgA1 glycoforms with some galactose-deficient (Gd) HR *O*-glycans play a key role in IgAN pathogenesis. The clustered and variable *O*-glycans make the IgA1 glycomic analysis challenging and better approaches are needed. Here, we report a comprehensive analytical workflow for IgA1 HR *O*-glycoform analysis. We combined an automated quantitative analysis of the HR *O*-glycopeptide profiles with sequential deglycosylation to remove all but Gd *O*-glycans from the HR. The workflow was tested using serum IgA1 from healthy subjects. Twelve variants of glycopeptides corresponding to the HR with three to six *O*-glycans were detected; nine glycopeptides carried up to three Gd *O*-glycans. Sites with Gd *O*-glycans were unambiguously identified by electron-transfer/higher-energy collision dissociation tandem mass spectrometry. Extracted ion chromatograms of isomeric glycoforms enabled quantitative assignment of Gd sites. The most frequent Gd site was T^236^, followed by S^230^, T^233^, T^228^, and S^232^. The new workflow for quantitative profiling of IgA1 HR *O*-glycoforms with site-specific resolution will enable identification of pathogenic IgA1 HR *O*-glycoforms in IgAN.

## Introduction

Glycosylation is an important post-translational modification that alters the structure and function of proteins^1^. Among immunoglobulins (Igs), the effect of the Asn^297^-linked *N*-glycan on IgG effector functions is well known. The different glycan structures affect antibody-dependent cellular cytotoxicity and complement-mediated cytotoxicity^2–4^. Furthermore, IgG glycosylation patterns are associated with various diseases, *e.g.*, reduced galactosylation and sialylation of IgG *N*-linked glycans are linked to rheumatoid arthritis and systemic lupus erythematosus^5–10^. Although the detailed structure and function of IgG Fc *N*-linked glycans have been elucidated, IgA1 clustered *O*-glycosylation remains to be comprehensively characterized.

**Figure 1:**
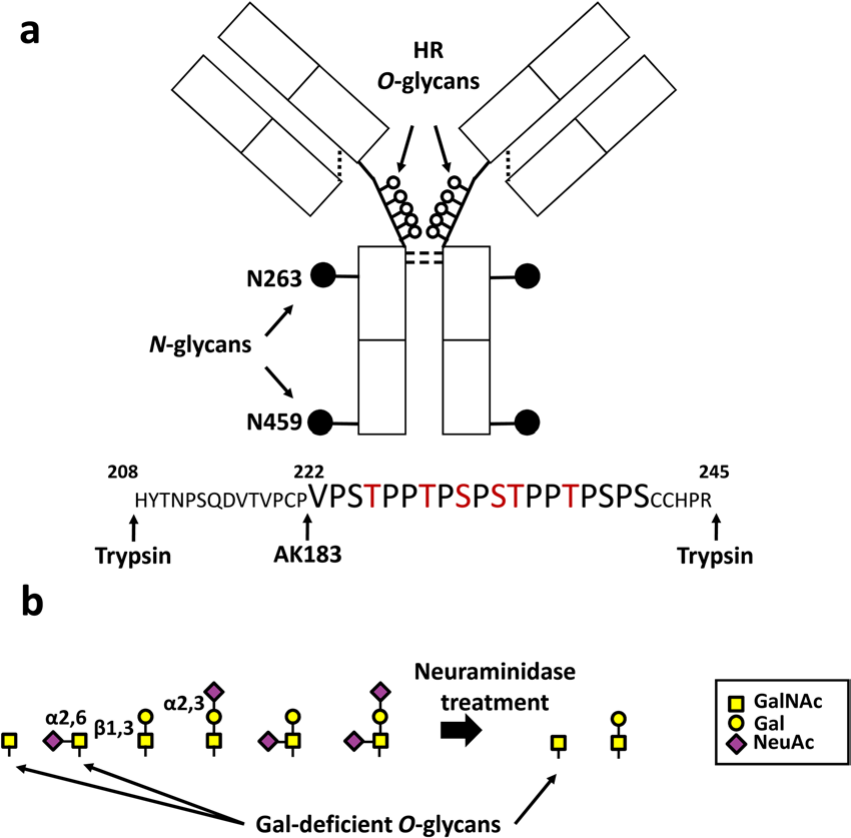
Human immunoglobulin A1 **(**IgA1) structure and the hinge-region (HR) *O*-glycans. (**a**) IgA1 structure. The major differences between IgA1 and IgA2 subclasses are in the HR. Human IgA1 HR is longer than that of IgA2, and with mucin-type clustered core 1 *O*-glycans attached (open circles). The HR usually has three to six *O*-glycan chains attached to some of the nine potential *O*-glycosylation sites. Six serine (S) and threonine (T) residues denoted in red are frequently glycosylated. (**b**) *O*-glycan variants of the serum IgA1. *N*-acetylgalactosamine (GalNAc) is attached to S/T residues in the HR. The *O*-glycan chain can be extended by the attachment of galactose (Gal) to GalNAc residues. GalNAc or Gal can be sialylated. Due to the step-wise nature of the *O*-glycosylation process, the IgA1-HR *O*-glycoforms exhibit a wide heterogeneity. An efficient neuraminidase treatment can reduce the complexity of IgA1-HR *O*-glycans to a mixture of disaccharides (Gal-GalNAc) and/or monosaccharides (GalNAc). NeuAc, *N*-acetylneuraminic acid.

IgA is the second most abundant antibody in the serum. Humans have two IgA subclasses, IgA1 and IgA2. Compared to IgA2 and other Igs, the structure of the hinge region (HR) of IgA1 is unique. The HR of IgA1 consists of a relatively long amino-acid sequence that includes nine serine (S) and threonine (T) residues, the potential *O*-glycosylation sites^11^. However, only three to six *O*-glycans are attached to IgA1 HR^12,13^ (Fig. 1). The *O*-glycans of IgA1 are core 1 glycans and are synthesized in a step-wise manner by a sequential action of several glycosyltransferases in the Golgi apparatus of the IgA1-secreting plasma cells^14^. First, *N*-acetylgalactosamine (GalNAc) residue(s) are attached to the S or T residues of the HR by polypeptide GalNAc-transferases (ppGalNAc-Ts). There are 20 ppGalNAc T isoforms in humans, and ppGalNAc T2 has been suggested to be an essential enzyme for the initiation of *O*-glycosylation of IgA1^15^. The *O*-glycan chain can be extended by the attachment of galactose (Gal) to the GalNAc residues by core 1 β1,3-galactosyltransferase. The glycan structure is completed by sialyltransferases that attach the negatively charged sialic acid to Gal or GalNAc residues (α2,3-linked to Gal and α2,6-linked to GalNAc)^16^. Hence, the IgA1 HR *O*-glycoforms can potentially be highly diverse in terms of the glycan attachment sites, the number of *O*-glycan chains, and the glycan structures.

Regarding the function of IgA1 HR *O*-glycans, HR *O*-glycans of secretory IgA1 (sIgA) serve as bacterial interaction sites, enabling IgA to participate in innate immunity in addition to adaptive immunity^17^. From that point of view, *N*- and *O*-glycosylation analysis of sIgA isolated from human colostrum and saliva was previously performed^18–20^. In serum IgA1, altered IgA1 *O*-glycosylation has been reported in pregnancy and several IgA-related diseases, including IgA nephropathy (IgAN) and IgA vasculitis with nephritis^21–24^. In IgAN, galactosylation of the HR *O*-glycoforms in the serum IgA1 and glomerular IgA1 is reduced. Consequently, the serum levels of IgA1 with Gal-deficient (Gd) *O*-glycans, with terminal or sialylated GalNAc residues, are elevated in IgAN^25–27^. These observations stem from a lectin-based enzyme-linked immunosorbent assay (ELISA) using *Helix aspersa* agglutinin (HAA), a lectin specifically recognizing terminal GalNAc. Furthermore, IgA1 in glomerular immunodeposits is enriched for Gd-IgA1 glycoforms, as revealed by matrix-assisted laser desorption ionization time of flight mass spectrometry (MALDI-TOF MS) and lectin ELISA^28,29^. There are some studies reporting that differential serum IgA1 HR *O-*glycosylation impacts pathogenesis of IgAN and disease progression. For example, hypo-galactosylated IgA may be prone to self-aggregation and, when recognized by autoantibodies specific for galactose-deficient IgA1 (Gd-IgA1), it forms immune complexes^30–32^. Furthermore, agalactosylated IgA1 has increased affinity for extracellular matrix proteins, such as fibronectin and type IV collagen^30–32^.

Recently, monoclonal antibodies (mAbs) specific for Gd-IgA1 were developed and their high reactivity with serum IgA1 from IgAN patients was demonstrated^33,34^. Furthermore, immunostaining with these mAbs revealed that the glomerular IgA1 in IgAN and IgA vasculitis with nephritis contain Gd-IgA1^35^. However, *O*-glycoforms of IgA1 in renal immunodeposits have not been characterized at the molecular level. This type of analysis to elucidate the *O*-glycan structure of IgA1 involved in the pathogenesis of IgAN will require not only quantitative *O*-glycopeptide profiling but also determination of Gd *O*-glycan attachment sites.

We have previously characterized IgA1 HR *O*-glycan micro-heterogeneity and attachment sites^36–38^. The current standard analysis of IgA1 *O*-glycan heterogeneity involves two steps: (1) profiling of IgA1 HR *O*-glycopeptides by liquid chromatography-high-resolution mass spectrometry (LC-HRMS) to identify IgA1 HR *O*-glycoforms; and (2) analyzing HR fragments generated with an IgA protease(s) by using electron-transfer dissociation (ETD) tandem MS to identify the sites of *O*-glycan attachment in individual IgA1 HR *O*-glycoforms. One limitation of this protocol is the throughput, as the Gd *O*-glycan attachment sites should be assigned in all *O*-glycoforms. In this study, we report a new workflow for a high-throughput glycomic analyses of IgA1 *O*-glycosylation. This workflow includes quantitative assessment of the attachment sites of Gd *O*-glycans in IgA1 HR.

## Results

### Development of a new protocol and bioinformatics solution for IgA1 HR *O*-glycoform profiling by sequential deglycosylation

**Figure 2:**
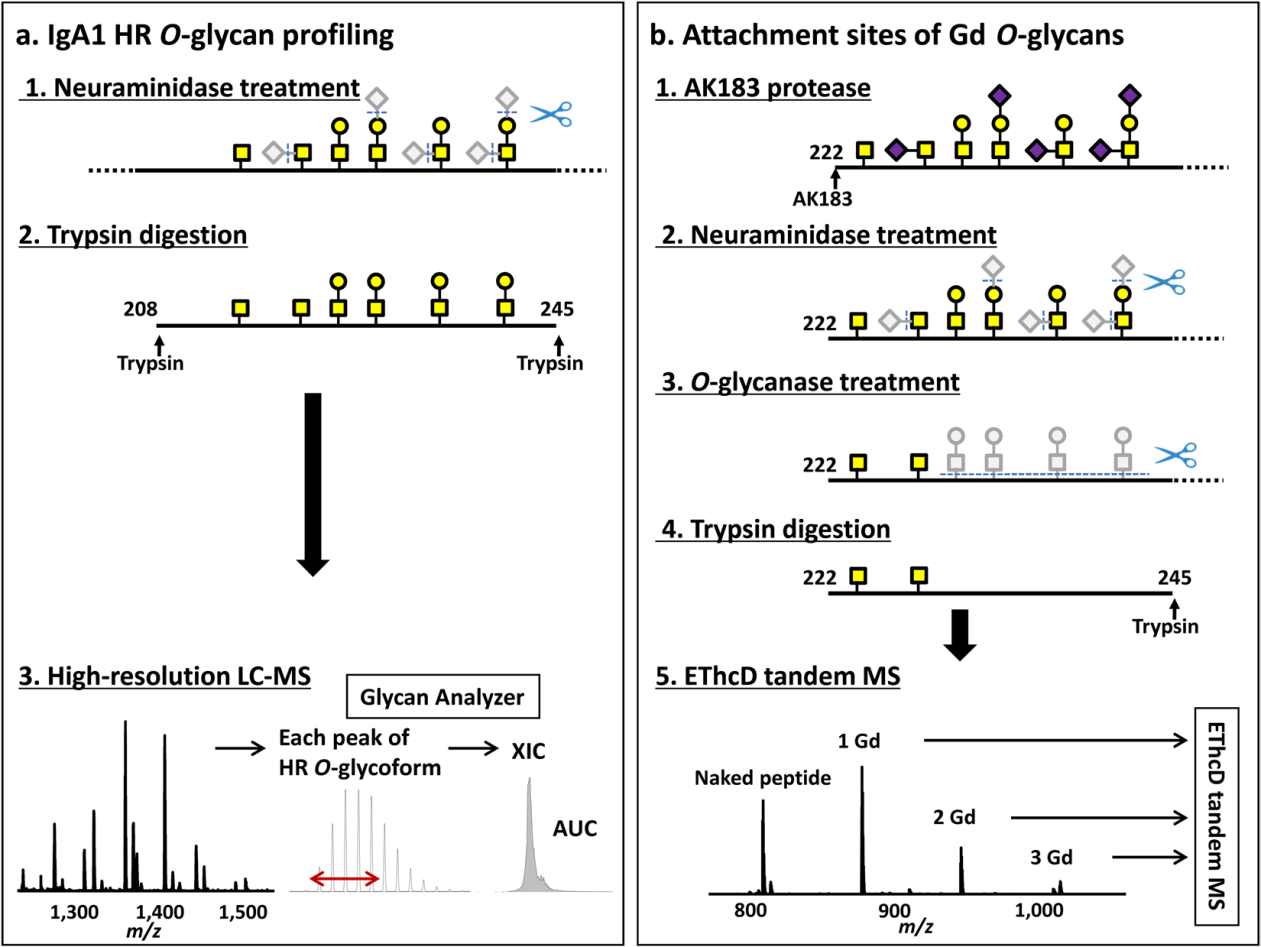
New workflow for IgA1 *O*-glycomic analysis by using the sequential deglycosylation protocol. (**a**) Protocol for the profiling of IgA1 HR *O*-glycans. Neuraminidase treatment reduces the types of *O*-glycan structures from six to two (disaccharide *N*-acetylgalactosamine [GalNAc]-galactose [Gal] and monosaccharide GalNAc). LC-MS spectrum contains several mass peaks of HR with *O*-glycans, according to the number of the attached GalNAc and Gal residues. An extracted ion chromatogram (XIC) is generated for each peak and the area under the curve (AUC) of XIC of each *O*-glycopeptide is determined and used to calculate the relative abundance (RA) of each *O*-glycoform (RA = percent of total XIC of *O*-glycoforms detected). (**b**) Protocol for the analysis of the attachment sites of galactose-deficient (Gd) *O*-glycan. IgA1 proteins were treated with an IgA-specific protease from *Clostridium ramosum* AK183. After a sequential enzymatic deglycosylation with neuraminidase and *O*-glycanase, that ultimately leaves only Gd *O*-glycans (*i.e*., GalNAc) attached to the amino-acid backbone, IgA1 is digested by trypsin. Precursor ions corresponding to HR with one to three Gd *O*-glycans are analyzed by LC-tandem MS with ETD combined with supplemental higher energy collision dissociation (HCD) activation (EThcD) to assign the attachment sites of Gd *O*-glycans.

To enhance throughput of IgA1 *O*-glycome quantitative profiling with site-specific resolution, we developed the sequential deglycosylation protocol. This protocol enables a quantitative removal of all but Gd *O*-glycans from the HR by using *O*-glycanase after neuraminidase treatment. To optimize efficiency of *O*-glycanase treatment, we compared two different *O*-glycanase preparations using polymeric IgA1 myeloma protein; one *O*-glycanase from *Streptococcus pneumoniae* and another one from *Enterococcus faecalis*. *O*-glycanase from *E. faecalis* showed superior efficacy, as only 0.42% of disaccharides remained on HR after the enzyme treatment, as compared to 63.94% for *S. pneumoniae* enzyme. (Supplementary Table S1). After the removal of all GalNAc-Gal disaccharides from IgA1, HR (glyco)peptides were produced by digestion with trypsin and analyzed by LC-HRMS with ETD combined with supplemental higher energy collision dissociation (HCD) activation (EThcD) to fragment HR Gd-glycopeptides to identify glycosylation sites (Fig. 2).

To increase the throughput of *O*-glycomic profiling of IgA1, we developed an in-house automated program, the Glycan Analyzer (MKI, Tokyo, Japan), to identify and quantify IgA1 *O*-glycoforms.

### MS profiling of IgA1 HR *O*-glycoforms

**Figure 3:**
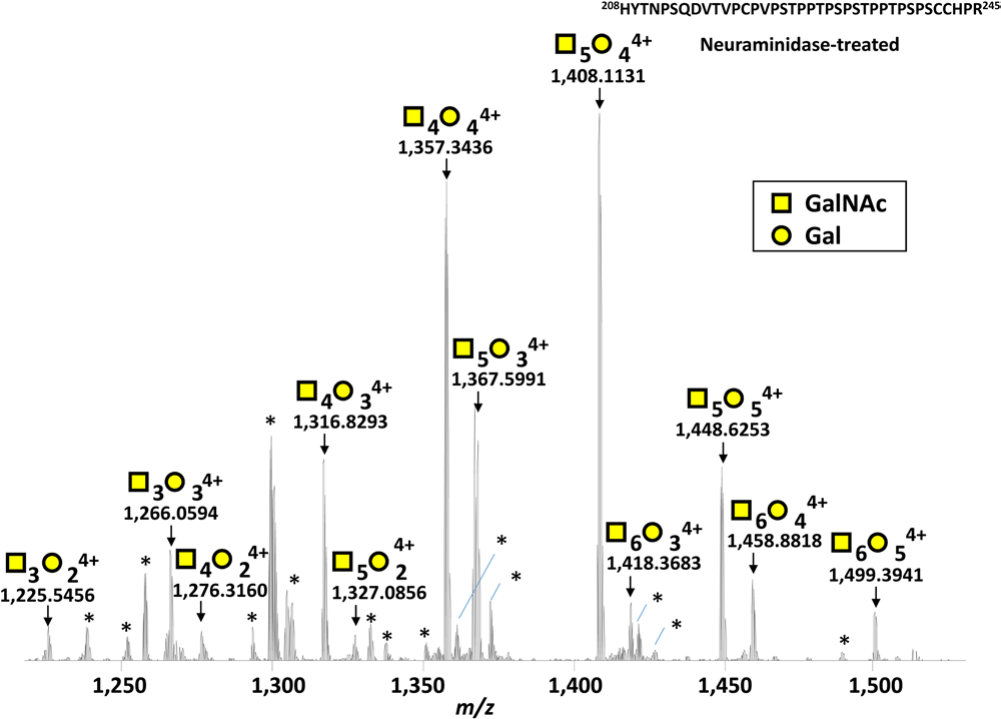
Representative mass spectrum of desialylated tryptic fragment of IgA1 HR *O*-glycoforms. The monoisotopic *m/z* value of HR *O*-glycopeptide ions, the number of sugar moieties assigned and their charge numbers are shown above the individual peaks. Twelve mass peaks of HR with *O*-glycans were detected, representing molecules with three to six *O*-glycan chains with up to three Gd *O*-glycans. *Marks unassigned peaks that were triply charged ions not (glyco)peptides derived from IgA1 HR (the detailed data are shown in Supplementary Fig. S4).

As susceptibility for IgAN is influenced by genetic background, and prevalence of IgAN is low in individuals of African ancestry, we used serum from healthy black subjects^39^. We have next tested our optimized bioinformatic solution by analyzing serum IgA1 isolated from 10 healthy subjects. Purified serum IgA1 was incubated with neuraminidase and then digested by trypsin. The neuraminidase treatment simplified the glycan structure and reduced the structural variety of attached glycans from six to two forms (*i.e.*, GalNAc and GalNAc-Gal; Fig. 1b). The resultant HR (glyco)peptides were then analyzed by using on-line LC-HRMS. Based on the potential number of GalNAc (*n* = 0–9, 203.0794 Da) and Gal (*n* = 0–9, 162.0528 Da) residues attached to HR (amino-acid sequence: His^208^–Arg^245^, 3,964.8182 Da), the presence of ions with theoretical masses of 55 potential *O*-glycopeptides was analyzed in all samples. Representative MS spectrum is shown in Fig. 3.

### Relative abundance of IgA1 HR *O*-glycoforms

Peak assignment and the area under the curve (AUC) of the extracted ion chromatogram (XIC) for each HR *O*-glycoform was accomplished by using an in-house developed software solution, the Glycan Analyzer. Twelve predominant HR *O*-glycoform variants were identified in all healthy subjects. All the HR glycopeptide variants were detected between min 14 and 18 of the LC analysis. The peaks assigned later than 18 min in Glycan Analyzer were identified as false-positive (f.p.). Furthermore, the assigned glycoforms with the main ions not being quadruply charged ions in Glycan Analyzer were also identified as false-positive. (The individual profiling and the summary of IgA1 HR *O*-glycoforms, determined by Glycan Analyzer and by manual procedure, are shown in Dataset 1 and 2, respectively).

**Figure 4:**
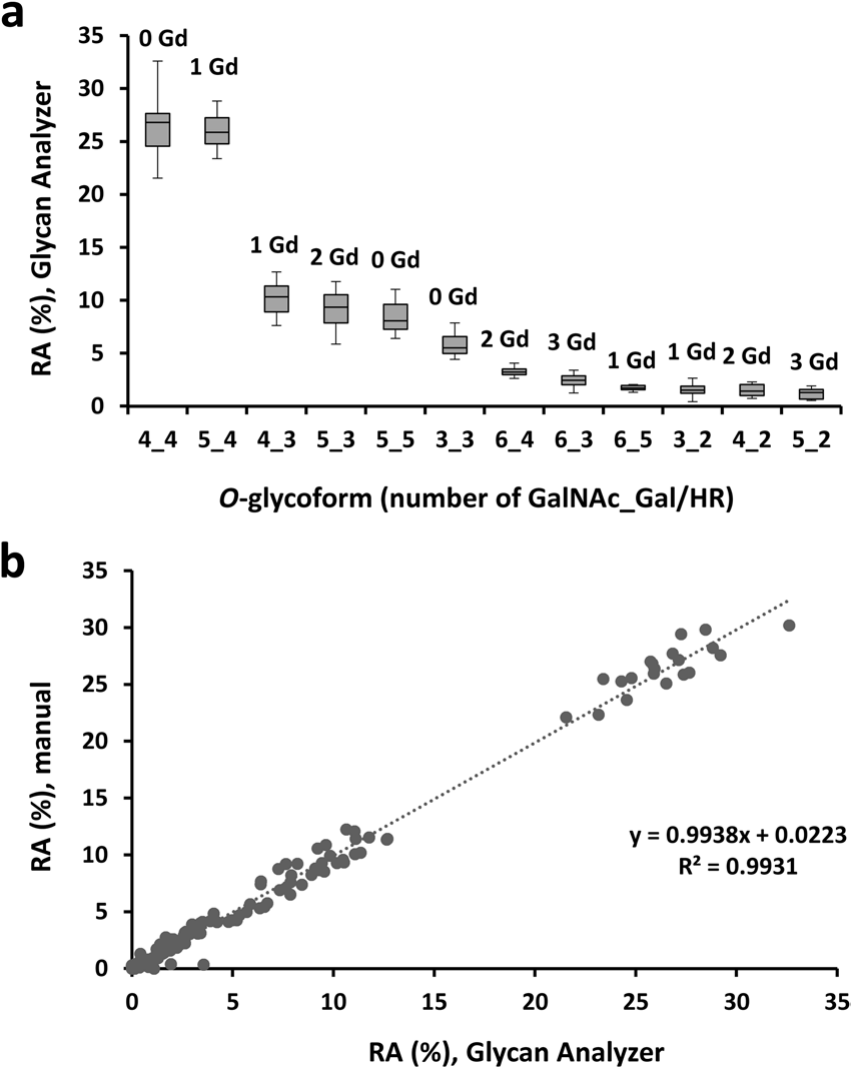
Relative abundance (RA) of various HR *O*-glycoforms in IgA1 isolated from sera of 10 healthy controls. (**a**) RA determined by using an automated program, Glycan Analyzer. RA of each *O*-glycopeptide is shown as a box plot with glycoforms on X axis in decreasing order of abundance. The number of attached GalNAc (x) and Gal (y) residues is notes as x_y on X axis. The number of Gd *O*-glycans in each *O*-glycopeptide is shown above the box. The upper and lower limits of the boxes represent the first and third quartiles of RA%. A segment inside the rectangle shows the median and the whiskers above and below the box show the minimum and maximum values. (**b**) Correlation between RA determined by Glycan Analyzer and by manual assessment. Linear regression analysis revealed a strong congruence between the two methods (y = 0.994x + 0.022, R² = 0.9931).

Relative abundance (RA) of each HR *O*-glycoform was expressed as a percentage of all *O*-glycoforms detected. RA [%, median, interquartile range (IQR)] of each *O*-glycoform in samples from 10 healthy subjects is shown in Fig. 4a and summarized in Table 1. HR glycoforms with 4 GalNAc and 4 Gal residues and HR with 5 GalNAc and 4 Gal residues were the two most abundant *O*-glycoforms (26.82% and 25.88%, respectively; P = 0.736). HR glycoforms with three to six *O*-glycan chains were observed; nine variants of Gd *O*-glycoforms with up to three Gd *O*-glycans were identified and designated as 1 Gd, 2 Gd, and 3 Gd. The median percentage of Gd *O*-glycan was 57.61% (54.17–61.95), on using Glycan Analyzer, and seemed to have some individual variation. To assess the performance of the automated analyses, we also performed manual analysis of the data and found that the RA values determined by Glycan Analyzer correlated well with the manually determined RA values (R^2^ = 0.993, P < 0.001) (Fig. 4b).

**Table 1:** The relative abundance (RA, %) for each *O*-glycopeptide.

Number of GalNAc_Gal/HR	RA determined by Glycan Analyzer [%, (IQR)]	RA determined manually [%, (IQR)]
	*n* = 10	*n* = 10
3_2	1.51	1.65
	(1.22–1.90)	(1.31–1.73)
3_3	5.51	4.84
	(4.98–6.58)	(4.25–5.43)
4_2	1.43	1.36
	(0.99–2.04)	(0.89–1.85)
4_3	10.32	9.43
	(8.91–11.35)	(8.25–10.20)
4_4	26.82	25.91
	(24.56–27.66)	(23.65–27.15)
5_2	1.3	1.2
	(0.66–1.59)	(0.73–1.38)
5_3	9.36	9.05
	(7.88–10.52)	(7.61–9.93)
5_4	25.88	26.92
	(24.79–27.25)	(25.56–28.20)
5_5	8.06	9.21
	(7.27–9.62)	(8.21–10.88)
6_3	2.45	2.53
	(2.03–2.87)	(2.26–3.06)
6_4	3.23	3.85
	(2.98–3.50)	(3.38–4.11)
6_5	1.71	2.21
	(1.56–1.97)	(1.98–2.58)

RA (%) of each *O*-glycopeptide generated by an automated program (Glycan Analyzer) or manually is shown. Data are expressed as the median (interquartile range: IQR) from 10 healthy controls.

### Quantitative assessment of Gd *O*-glycan sites in IgA1 HR

**Figure 5:**
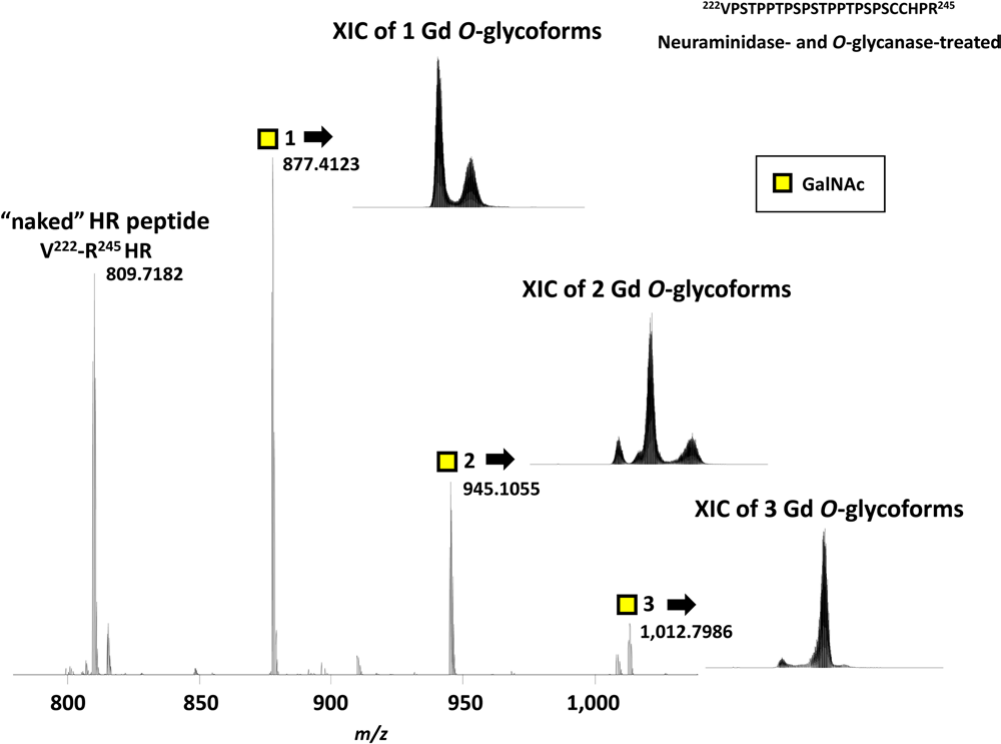
Mass spectrum and extracted ion chromatogram (XIC) of IgA1 HR peptide and glycopeptides with Gd *O*-glycans after sequential deglycosylation. Sequential neuraminidase and *O*-glycanase treatment leaves only Gd *O*-glycan(s) at V^222^–R^245^ HR. The respective peaks correspond to naked V^222^–R^245^ HR and V^222^–R^245^ HR with one, two, or three Gd *O*-glycans. The number of GalNAc residues attached to HR is designated by the number after yellow squares. The theoretical monoisotopic mass values of “naked” V^222^–R^245^ HR peptide and V^222^–R^245^ HR glycopeptide with one to three Gd *O*-glycans are shown beside the peaks. The XIC for each Gd *O*-glycoform is shown next to the respective ion peak.

**Figure 6:**
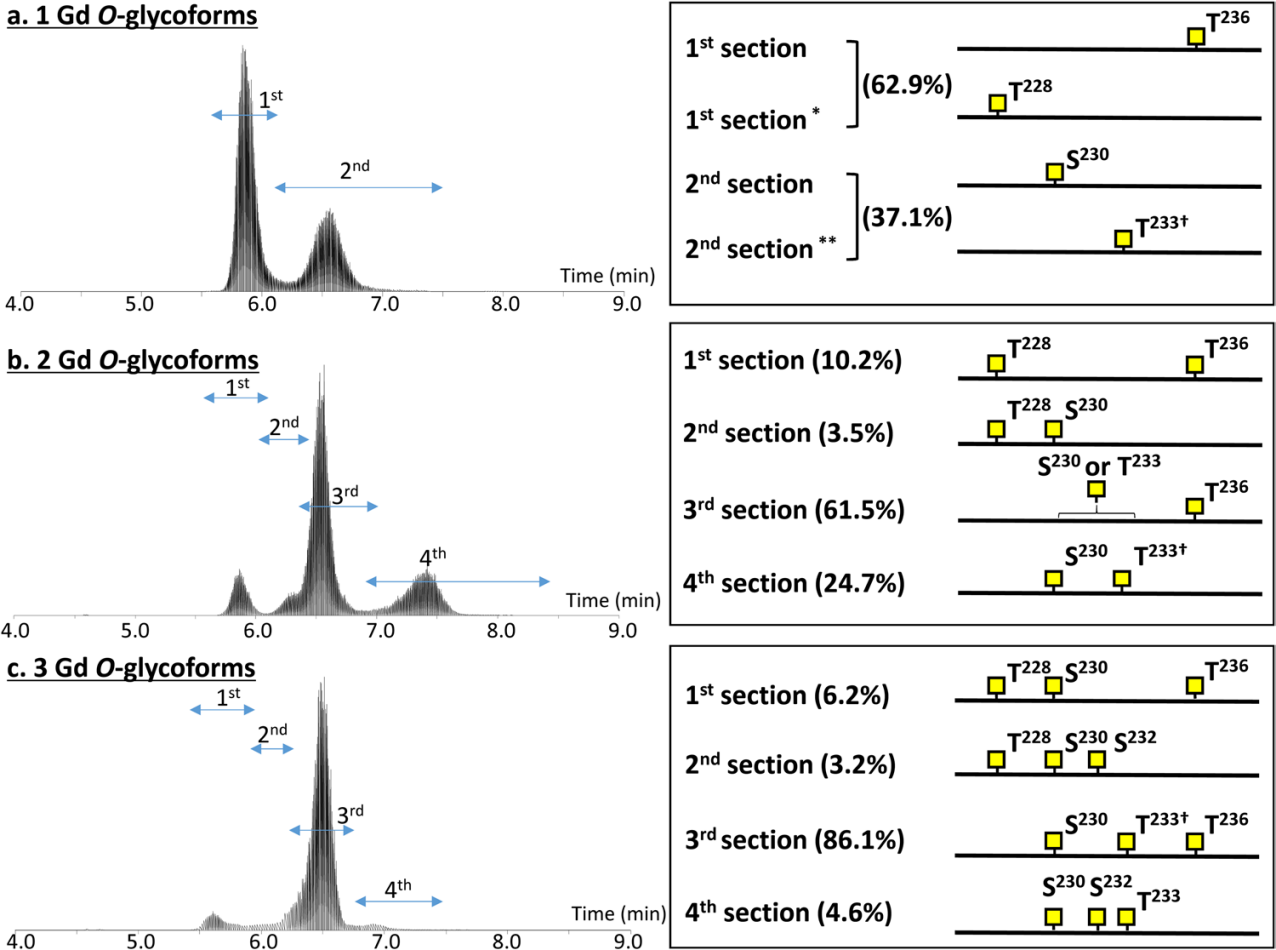
Summary of Gd *O*-glycan sites in the serum IgA1 HR identified by EThcD-tandem MS. (**a**) The extracted ion chromatogram (XIC) and attachment sites of Gd *O*-glycan in 1 Gd *O*-glycoform. (**b**) The XIC and attachment sites of Gd *O*-glycans in 2 Gd *O*-glycoform. (**c**) The XIC and attachment sites of Gd *O*-glycans in 3 Gd *O*-glycoform. The XIC is divided into several sections and the attachment sites of Gd *O*-glycans are shown individually. The XIC of product ions are shown in Supplementary Figs. S1–S3. Relative abundance of each isomeric glycoform is shown as the percentage of total Gd *O*-glycoforms. ^*^Subsection of the 1^st^ section, ^**^Subsection of the 2^nd^ section. ^†^*O*-glycoform includes a small amount of isomers with Gd *O*-glycan at S^232^ instead of T^233^. The XIC of each precursor ion (H^208^-R^245^ HR plus 1–3 Gd *O*-glycan) detected from five individual sample named (BM03, BM04, BW00, BW01, and BW02) are available in Supplemental Fig. S5. Relative abundance of each isomeric glycoform of individual samples are shown in Dataset 3.

After removal of disaccharides (GalNAc-Gal) from HR by *O*-glycanase, the number of IgA1 HR (glyco)peptides was reduced from 12 to 4; these HR variants corresponded to the naked (*i.e.*, fully deglycosylated) HR peptide, and HR glycopeptides with one to three Gd *O*-glycans (Fig. 5). The XIC of each precursor ion is shown in Fig. 5. RA of each *O*-glycopeptide is listed in Supplementary Table S2. Three trivalent cations corresponding to V^222^–R^245^ HR with one Gd *O*-glycan (1 Gd *O*-glycoform, *m/z* 877.4123), HR with two Gd *O*-glycans (2 Gd *O*-glycoform, *m/z* 945.1055), and HR with three Gd *O*-glycans (3 Gd *O*-glycoform, *m/z* 1,012.7986) were analyzed by EThcD tandem MS to identify the site(s) of glycan attachment. To identify the glycan attachment site(s), the presence or absence of *c* ions with 0–3 GalNAc and *z* ions with 0–3 GalNAc was determined based on the theoretical product ion mass (Supplementary Tables S3–S5). To identify potential isomeric structure(s) based on the alternative attachment sites of the GalNAc residue(s)^38^, the detection time of each product ion was determined by generating the XIC (Supplementary Figs. S1–S3). After assigning the glycan attachment sites, XIC of each Gd *O*-glycopeptide isomer was analyzed and expressed as the percentage against all Gd *O*-glycopeptides (Fig. 6).

The XIC of 1 Gd *O*-glycoform (*m/z* 877.4123) showed a bimodal peak (Fig. 6a). EThcD fragmentation clearly indicated that 1 Gd *O*-glycoform eluting early (section 1 in Fig. 6a, RT 5.5–6.2 min; 62.9% of 1 Gd *O*-glycoform) had Gd *O*-glycan attached mostly at T^236^ and partially at T^228^. For the late-eluting isomers (section 2 in Fig. 6a, RT 6.2–13.9 min), 1 Gd *O*-glycan was mainly attached at S^230^ and partially at T^233^ or, to a lesser extent, at S^232^. Hence, the most frequent site of Gd *O*-glycan attachment in 1 Gd *O*-glycoform is T^236^, followed by S^230^, T^233^, T^228^, and S^232^. The XIC of 2 Gd *O*-glycoform (*m/z* 945.1055) was divided into four sections (Fig. 6b). The predominant form of 2 Gd *O*-glycoform (61.5%) had Gd *O*-glycans at T^236^ and S^230^ or T^233^. Furthermore, 2 Gd *O*-glycoforms with Gd *O*-glycan at T^228^ and T^236^ or S^230^ were detected in early sections (sections 1 and 2 in Fig. 6b, RT 5.5–6.3 min). Another 2 Gd *O*-glycoform, eluting later (section 4 in Fig. 6b, RT 6.7–8.5 min), had Gd *O*-glycans attached at S^230^ and T^233^. The XIC of the precursor ion of 3 Gd *O*-glycoform (*m/z* 1,012.7986) was divided into four sections (Fig. 6c). The predominant form of 3 Gd *O*-glycoform (86.1%) had Gd *O*-glycans at S^230^, T^233^, and T^236^. In the early sections (sections 1 and 2 in Fig. 6c, RT 5.4–6.1 min), Gd *O*-glycans of the 3 Gd *O*-glycoforms were located at T^228^, S^230^, and T^236^ or S^232^. In the late section (section 4 in Fig. 6c, RT 6.7–8.5 min), Gd *O*-glycans were attached in the central region of HR, *i.e.*, at S^230^, S^232^, and T^233^. Based on these observations, we conclude that the Gd *O*-glycans were most frequently attached at T^236^, followed by S^230^, T^233^, T^228^, and S^232^.

## Discussion

In the current study, we devised a novel protocol for quantitative and qualitative profiling of Gd *O*-glycans in the IgA1 HR. We then used this protocol to successfully profile *O*-glycans in the IgA1 HR in healthy individuals, laying groundwork for studies of IgA1-related pathologies.

As determined by anti–Gd-IgA1 mAbs and GalNAc-specific lectins, the serum levels of Gd-IgA1 are elevated in individuals with IgAN and glomerular immunodeposits in these individuals are enriched for Gd-IgA1. As anti–Gd-IgA1 mAbs recognize Gd *O*-glycans located only at a specific site(s), quantitative assessment of Gd sites is needed to identify IgA1 HR *O*-glycoforms that are disease-specific. Because *O-*glycan synthesis occurs in a step-wise manner, *O*-glycoforms in the IgA1 HR exhibit wide diversity with respect to the attachment site, the number of *O*-glycan chains, and *O*-glycan structure (Fig. 1). Such diversity generates analytical complexity. To address this issue, we developed a deglycosylation approach to simplify the *O*-glycoform structure before analysis. The ultimate application of the method is determining the differences in the amount of Gd *O*-glycans and their attachment sites in IgA1 from patients with IgA1-related disease, such as IgAN, and control individuals. This will only be possible by using a high-throughput protocol focusing on Gd *O*-glycans. Indeed, the protocol for quantitative profiling of IgA1 HR *O*-glycoforms devised in the current study reduced the structural variety of *O*-glycans from six to two structures (Fig. 1) and the neuraminidase treatment incorporated in the protocol enabled HR *O*-glycopeptides profiling (the number and structure of the *O*-glycan chains). Furthermore, Glycan Analyzer, an in-house developed automated program, assisted the high-throughput quantitative analysis of the IgA1 HR *O*-glycoforms.

To investigate the attachment sites of Gd *O*-glycans, we analyzed all (nine) HR *O*-glycoforms, including Gd *O*-glycans, by EThcD tandem MS to localize the Gd *O*-glycan attachment sites (Fig. 4a). Previously, we used three IgA-specific proteases (from *Clostridium ramosum* AK183, *Haemophilus influenzae* HK50, and *Streptococcus pneumoniae* TIGR4), to shorten the HR peptide, and analyzed the C-terminal and N-terminal glycopeptides in each digest, because an extensively long backbone peptide and clustered *O*-glycans hamper EThcD fragmentation, most likely because of insufficient charge density^36,37^. In the current workflow, the *O*-glycanase digestion removed all but Gd *O*-glycans from the HR (Fig. 2), reducing the minimum number of target precursor ions and enhancing their intensity for EThcD tandem MS. This modification allowed a successful quantitative assessment of the Gd *O*-glycan attachment sites in a single preparation of IgA1 HR.

The attachment sites of *O*-glycans in circulating IgA1 were determined by Mattu *et al*.^12^ to be T^225,^ T^228^, S^230^, S^232^, and T^236^. Five *O*-glycosylation sites in IgA1 myeloma proteins, Gd-IgA1 mimics, have been reported^37^ the frequent sites of Gd attachment are S^230^ and T^236^. We further identified a sixth native site of *O*-glycosylation at T^233^, as well as isomers with different glycan positions and glycan-chain structures^38^. Our EThcD fragmentations of 1, 2, and 3 Gd *O*-glycoforms clearly showed that the most frequent site for Gd *O*-glycans was T^236^, followed by S^230^, T^233^, T^228^, and S^232^. T^225^ was not a site for Gd *O*-glycan in IgA1 from any healthy individual in this study. Gal deficiency occurs mainly in the positions previously reported to be variably glycosylated and it is rare at sites that are consistently predominantly glycosylated. KM55, a mAb recently used for detection of Gd-IgA1, recognizes T^225^ and/or T^233^ in synthetic HR peptides^40^. However, it remains unclear whether this antibody recognizes a specific stereoscopic structure(s) of Gd-IgA1 with Gal-deficient sites at T^225^ and/or T^233^. Comparison of serum level of Gd-IgA1 measured by Gd-IgA1 mAb ELISA and glycoprofiling generated by our new workflow would help identify specific IgA1 *O*-glycoforms elevated in IgAN. Furthermore, we can directly detect the specifically elevated Gal-deficient site(s) in IgA1 from IgAN patients by comparing the XIC of the three precursor ions in our approach between healthy subjects and IgAN patients.

In the current study, we identified 12 *O*-glycoforms of the desialylated serum IgA1 HR from healthy subjects, with three to six *O*-glycan chains attached per HR. The frequently occurring *O*-glycoforms were 4 GalNAc 4 Gal and 5 GalNAc 4 Gal, followed by 4 GalNAc 3 Gal, 5 GalNAc 3 Gal, 5 GalNAc 5 Gal. The most abundant Gd *O*-glycoform was 1 Gd *O*-glycoform, which accounted for approximately 40% of IgA1 (Table 1 and Supplementary Table S2). By using on-line LC EThcD tandem MS, we elucidated the isomers of Gd *O*-glycan attachment sites for the 1–3 Gd *O*-glycopeptides. The main sites for Gd *O*-glycan attachment in 1 Gd *O*-glycoform were T^236^ and S^230^. The XIC of 1 Gd *O*-glycoform showed a bimodal peak. The early peak, accounting for 62.9% of 1 Gd *O*-glycoform, mostly contained *O*-glycoforms with Gd *O*-glycan attached at T^236^ and the late peak, accounting for 37.1% of 1 Gd *O*-glycoform, mostly contained molecules with *O*-glycan at S^230^. The Gd *O*-glycoforms with *O*-glycan attached at T^228^ and T^236^ were generally detected in the early phase of LC, as shown in Fig. 6. Based on the properties of reversed-phase LC, the glycopeptides eluting in the early phase are more hydrophilic. A proline-rich segment (the PPTP motif) is present in the vicinity of residues T^228^ and T^236^. Because proline is a hydrophobic amino acid, the attachment of GalNAc to T^228^ and T^236^ is highly likely to change the hydrophobic nature of HR, to a more hydrophilic nature. Furthermore, proline is thought to impact the protein architecture because its ring structure renders it more conformationally restricted than the other amino acids. Proline has a much greater propensity than other naturally occurring amino acids to form *cis*-amide bonds^41^. By contrast, a glycosylated S/T residue frequently alters the conformation of C-terminal proline to a *trans* conformation^42^. The attachment of GalNAc to T^228^ and T^236^ may also be important for the alteration of protein hydrophilic-hydrophobic nature and architecture for serum IgA1.

As a limitation of the developed method, we were not able to detect the attachment sites of galactosylated *O*-glycan chains. However, the pathogenesis of IgAN probably involves a specific Gd site^40^. In the context of investigating disease-specific Gd sites, removal of galactosylated *O*-glycans by sequential deglycosylation represents a considerable advantage for detecting the Gd *O*-glycan attachment sites. Furthermore, the function of proteins with clustered *O*-glycans,* e.g.*, immunoglobulins, mucins, and some bacterial cell-surface glycoproteins, is under-investigated because of the glycoprotein complexity. The protocol presented in the current study lays the framework for a successful definition of the attachment sites and overall microheterogeneity of such proteins.

In conclusion, the new workflow, involving a high-resolution LC-MS with ETD tandem MS following a removal of galactosylated *O*-glycans, could be a powerful tool for the identification of specific glycoforms and specific Gd *O*-glycan attachment sites in IgA1 of individuals with IgAN.

## Materials and Methods

### Ethical approval

All experimental protocols were approved by Institutional Review Board (IRB) of Fujita Health University (approved number HM18-421). Informed consent was obtained from all subjects, and all methods were performed in accordance with the relevant guidelines and regulations. Serum sample collected from consented healthy donors under IRB-approved protocols in use at FDA-licensed donor centers (Bioreclamation IVT, Westbury, NY).

### Purification of IgA1 and sample preparation for MS analysis

Polymeric IgA1 (Mce1) myeloma protein was isolated from plasma of a patient with IgA1 myeloma, as described previously^38^. IgA1 was purified from 100 μl of serum of 10 black healthy subjects (30249993, Bioreclamation IVT, Westbury, NY) by using affinity chromatography with anti-human IgA (0855068, Cappel Laboratories, Cochranville, PA, USA) coupled to a cyanogen bromide-activated Sepharose column. Purified samples were stored aliquoted at −80 °C^38^. For IgA HR *O*-glycoform profiling, 5 μg of purified IgA1 proteins were treated with 2.5 mU neuraminidase (GK80040, ProZyme, Hayward, CA, USA) in 50 mM sodium phosphate, pH 6.0, at 37 °C, overnight. To detect Gd *O*-glycan attachment sites, purified IgA1 proteins were treated with an IgA-specific protease from *Clostridium ramosum* AK183 (a recombinant enzyme produced in *Escherichia coli*)^37^. Then, 5 μg of digested protein were treated with 2.5 mU neuraminidase. To remove disaccharides from HR glycopeptides, the digests were treated overnight with 40,000 U *O*-glycanase from *Enterococcus faecalis* (P0733, New England BioLabs, Ipswich, MA, USA) at 37 °C. To compare deglycosylation efficacy, 40,000 U *O*-glycanase from *Enterococcus faecalis* and 1.25 mU *O*-glycanase from *Streptococcus pneumoniae* (GK80090, Agilent, Santa Clara, CA, USA) were tested using polymeric IgA1 myeloma protein after neuraminidase treatment. Before MS analysis, disulfide bridges in the samples were reduced by incubating with 20 mM DTT for 15 min at room temperature and the samples were digested by trypsin (GK80040, Promega, Madison, WI, USA) (enzyme-to-substrate ratio of 1:50) in 100 mM NH_4_HCO_3_, pH 8.3, at 37 °C, overnight. The digests were desalted by passing through C18 spin columns (89873, Thermo Fisher Scientific, San Jose, CA, USA) and the solvent was removed by evaporating using a SpeedVac.

### LC-MS analysis for profiling of IgA HR *O*-glycoforms

On-line LC was performed using an EASY-nLC 1000 system (Thermo Fisher Scientific). For the analysis, 500 ng of desialylated and trypsin-digested IgA1 was loaded onto C18 EASY-Spray column (75 μm × 15 cm, 2.1 μm, 100 Å, Thermo Fisher Scientific). The digests were eluted using an acetonitrile gradient from 0% to 35% in 0.1% formic acid over 30 min at a flow rate of 300 nl/min. Hybrid quadrupole mass filter/linear ion trap/orbitrap MS (Orbitrap Fusion, Thermo Fisher Scientific) was alternated between a full orbitrap MS scan (*m/z* 500–1,700) at a resolving power of 120,000, the S-lens radio frequency level set at 60%, and a subsequent MS/MS scan of the abundant precursor ions.

### EThcD tandem MS analysis for the detection of Gd *O*-glycan attachment sites

After a sequential treatment of IgA1 by the IgA-specific protease (AK183), neuraminidase, *O*-glycanase, and trypsin (as described above), a desalted preparation corresponding to 2 μg of a mixture of IgA1 from 5 subjects was loaded onto on-line LC system for each target precursor ion measurement. The samples were eluted using an acetonitrile gradient from 11% to 18% in 0.1% formic acid over 20 min, at a flow rate of 300 nl/min. The mass spectrometer was set to target mass mode to automatically switch between MS and MS/MS acquisition. Full-scan MS spectra (*m/z* 780–1,200) were acquired using the Orbitrap analyzer at a resolution of 120,000, with a target value of charges (automatic gain control) of 4 × 10^5^.

For EThcD experiments, three trivalent cations corresponding to HR with one Gd *O*-glycan (*m/z* 877.7456), HR with two Gd *O*-glycan (*m/z* 945.4388), and HR with three Gd *O*-glycan (*m/z* 1,013.1319) were set as precursor ions. These ions were isolated by using an isolation window (*m/z* 3) and subsequently subjected to ETD. ETD reaction time was set to 50 ms, ETD reagent target value was set to 1 × 10^6^, and the maximum ETD reagent injection time was set to 200 ms. ETD supplemental activation collision energy type was EThcD (25% for HR with one GalNAc residue, 28% for HR with two GalNAc residues, 32% for HR with three GalNAc residues). Although three fragmentation approaches, *e.g.*, ETD only, EThcD, or ETciD, were tested for HR and 1–3 Gd *O*-glycoforms, we could confirm sufficient fragmentation only by EThcD. This is similar to the original analysis of IgA1 *O*-glycsylation by activated ion-electron capture dissociation (AI-ECD) where sufficient electron radical dissociation was only observed with supplemental activation by an infrared laser^37,38,43^. The ETD reaction time and supplemental activation energy were also optimized for more efficient dissociation of the glycopeptides. Product ions were detected in the mass rage of *m/z* 200–3,000 by using the Orbitrap mass analyzer at a resolution of 15,000. An automatic gain control value for the precursor ions was 2 × 10^5^ and the maximum injection time was 1.

### Data analysis for IgA HR *O*-glycoform profiling

All spectra were analyzed by using Xcalibur Qual Browser 2.2 (Thermo Fisher Scientific) software. Individual IgA1 *O*-glycopeptides were identified as previously described^36–38,44^. Briefly, IgA1 *O*-glycopeptide species from each LC-MS analysis were identified by referencing the theoretical monoisotopic mass list, which was created based on mass values of trypsin-digested IgA1 HR amino acid sequences, and the number of attached GalNAc and Gal residues by using the GlycoMod tool (http://www.expasy.org). The minimal threshold for IgA1 HR glycopeptide identification was a signal-to-noise ratio of at least 5:1 with >5 isotopic peaks. The tolerance for glycopeptide identification was ±5 ppm. The relative quantitative distributions of each glycopeptide series were calculated by using a label-free method, as previously described^37,38,45^. The ion chromatogram was extracted from five isotopic peaks of each glycopeptide ion and the AUC was obtained. The RA (%) for each glycopeptide was obtained by dividing the AUC of each glycopeptide XIC by the total AUC for all glycopeptide XIC.

To increase the throughput of the analysis, an in-house automated program, Glycan Analyzer (MKI, Tokyo, Japan, https://www.mki.co.jp/english/bioinformatics.html), was developed. The program can be used to measure the AUC of each glycopeptide XIC automatically. Glycan Analyzer calculates theoretical mass values of Core 1 *O*-glycopeptide based on the masses of amino-acid sequence of HR plus the number of *N*-acetylhexosamine (*i.e.*, GalNAc), hexose (*i.e.*, Gal), and *N*-acetylneuraminic acid. The program read the raw file and assigned HR *O*-glycoforms within 5 ppm resolution. XIC of assigned *O*-glycoform was generated and the area under the curve was calculated on the program.

### Data analysis for detecting Gd *O*-glycan attachment sites

All spectra were analyzed using Xcalibur Qual Browser 2.2 (Thermo Fisher Scientific) software. First, XIC of each precursor ion was obtained from the full-mass scan. Theoretical lists of IgA1 HR peptides fragmented by ETD were generated by using the ProteinProspector MS product tool (http://prospector.ucsf.edu/), with the inclusion of *c* and *z* ions (Supplementary Tables S1–S3)^38^. To define the time range within which each product ion was detected, the ion chromatograms were extracted automatically by entering the mass range (theoretical *m/z* ± 10 ppm) of each product ion to Xcalibur Qual Browser system. The attachment sites of Gd *O*-glycans were manually assigned based on the combination of *c* ions and *z* ions.

After determining the attachment sites, the AUC from the precursor ion XIC at the time at which each Gd *O*-glycopeptide isomer was detected was measured. The percentage against total AUC of precursor ion XIC was expressed as RA.

### Statistics

Statistical analysis was performed using Statistical Package for the Social Sciences (SPSS) Statistics, Version 22.0 for Windows (IBM, Armonk, NY, USA). Continuous variables are shown as the median and IQR. Differences of the average values of 4 GalNAc 4 Gal and 5 GalNAc 4 Gal were tested by paired t-test because they were normally distributed. The relationship between the RA values calculated using the Glycan Analyzer or manually was evaluated by using linear regression analysis. P-values of <0.05 are considered to be statistically significant.

## Supplementary information

Supplementary figures and tables.

Dataset 1.

Dataset 2.

Dataset 3.

## Data Availability

All data generated during this study are available in Supplementary Dataset.
