# Author Correction: Analysis of *O*-glycoforms of the IgA1 hinge region by sequential deglycosylation

**DOI:** 10.1038/s41598-021-00847-w

**Published:** 2021-10-21

**Authors:** Yukako Ohyama, Hisateru Yamaguchi, Kazuki Nakajima, Tomohiro Mizuno, Yukihiro Fukamachi, Yasuto Yokoi, Naotake Tsuboi, Daijo Inaguma, Midori Hasegawa, Matthew B. Renfrow, Jan Novak, Yukio Yuzawa, Kazuo Takahashi

**Affiliations:** 1 Department of Nephrology, Fujita Health University School of Medicine, Toyoake, Japan; 2 Institute for Comprehensive Medical Science, Fujita Health University, Toyoake, Japan; 3 Center for Research Promotion and Support, Fujita Health University, Toyoake, Japan; 4 Analytical Pharmacology, Faculty of Pharmacy, Meijo University, Nagoya, Japan; 5 Mitsui Knowledge Industry, Tokyo, Japan; 6 Departments of Biochemistry and Molecular Genetics and Microbiology, University of Alabama at Birmingham, Birmingham, AL USA; 7 Department of Biomedical Molecular Sciences, Fujita Health University School of Medicine, Toyoake, Japan

Correction to: *Scientific Reports* 10.1038/s41598-020-57510-z, published online 20 January 2020

The Competing interests section in this Article was incomplete.

“The authors declare no competing interests.”

now reads:

“M. B. Renfrow and J. Novak are co-founders and co-owners of and consultants for Reliant Glycosciences, LLC and co-inventors on the US patent application 14/318,082 (assigned to UAB Research Foundation that distributes royalties to the inventors). The other authors declare no competing interests.”

The original Article has been corrected.

