# Supplementary material for: Analysis of O-glycoforms of the IgA1 hinge region by sequential deglycosylation
Source: Sci Rep. 2020 Jan 20;10:671. doi: 10.1038/s41598-020-57510-z (PMC6971281; doi:10.1038/s41598-020-57510-z)
Supplement: Supplementary file 1 — Supplementary figures and tables. [file 41598_2020_57510_MOESM1_ESM.pdf]

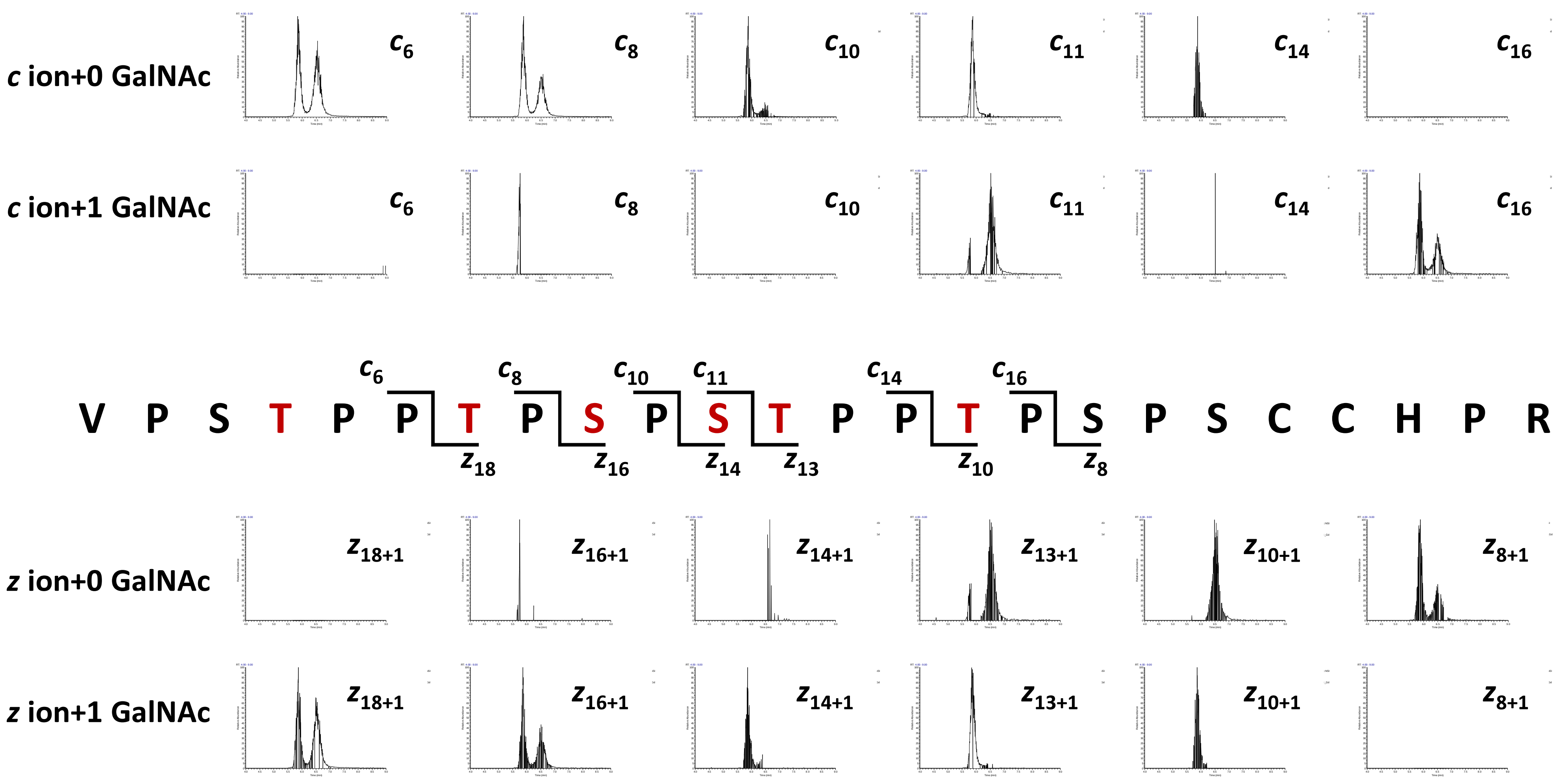

Supplementary Figure S1

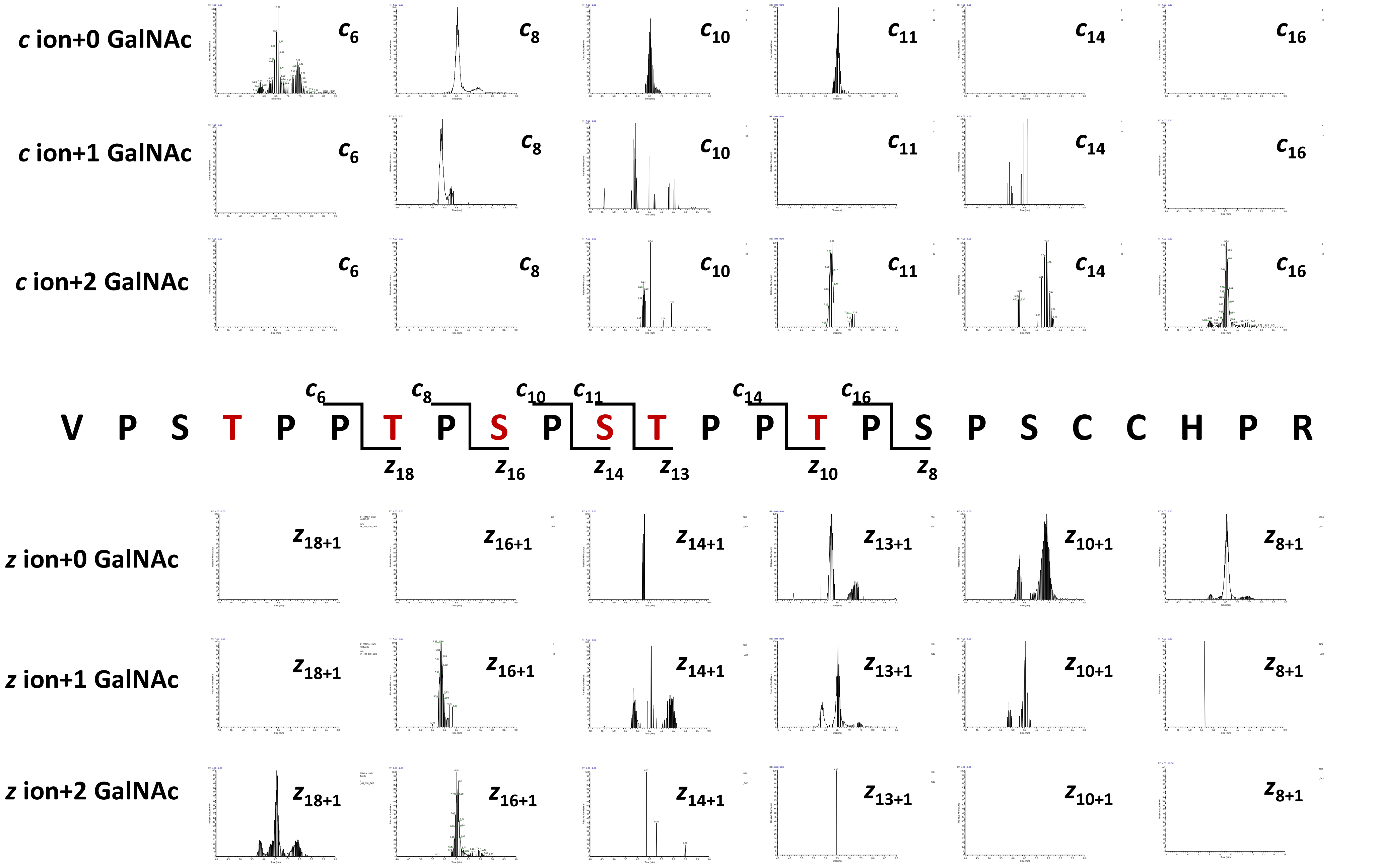

Supplementary Figure S2

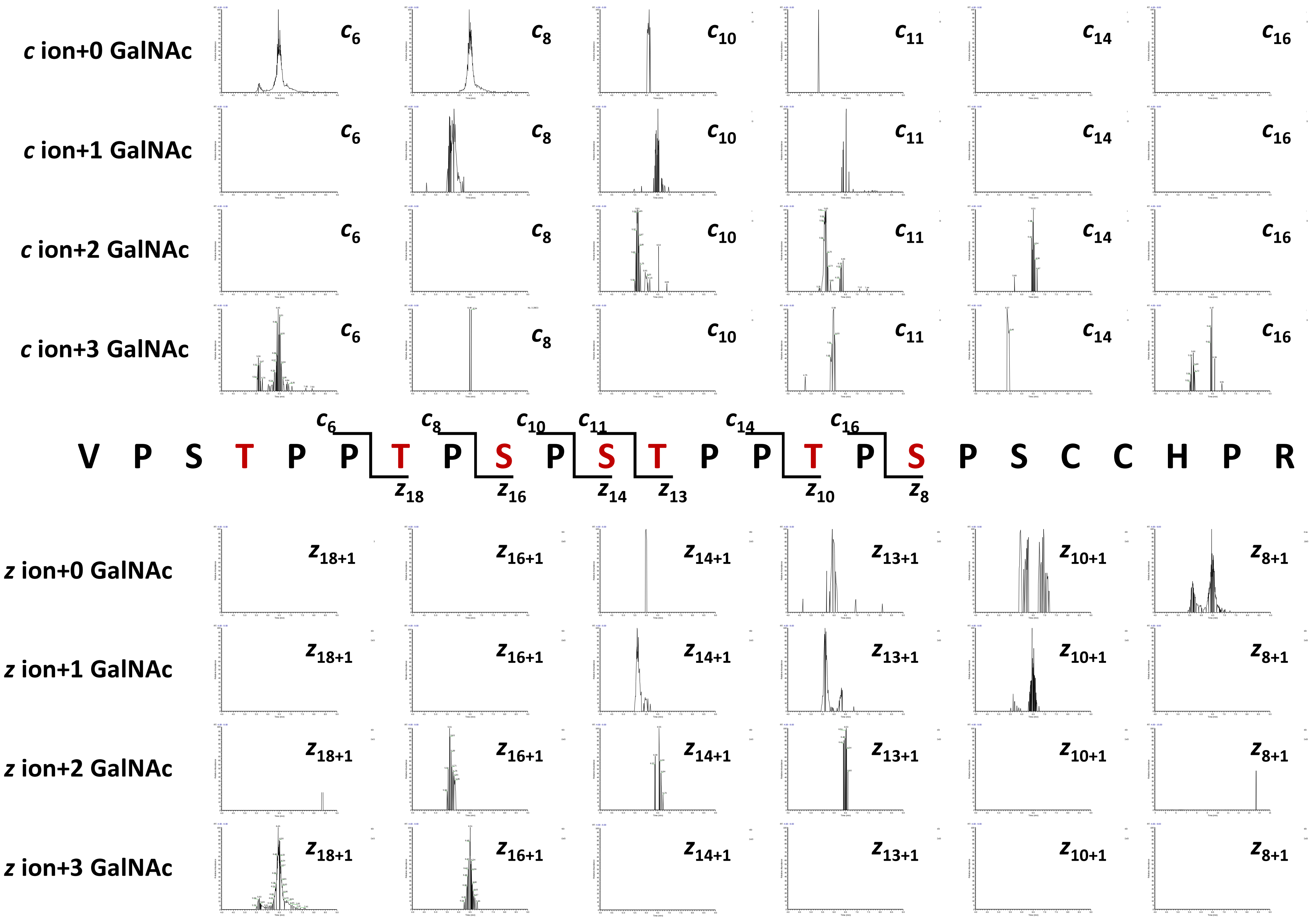

Supplementary Figure S3

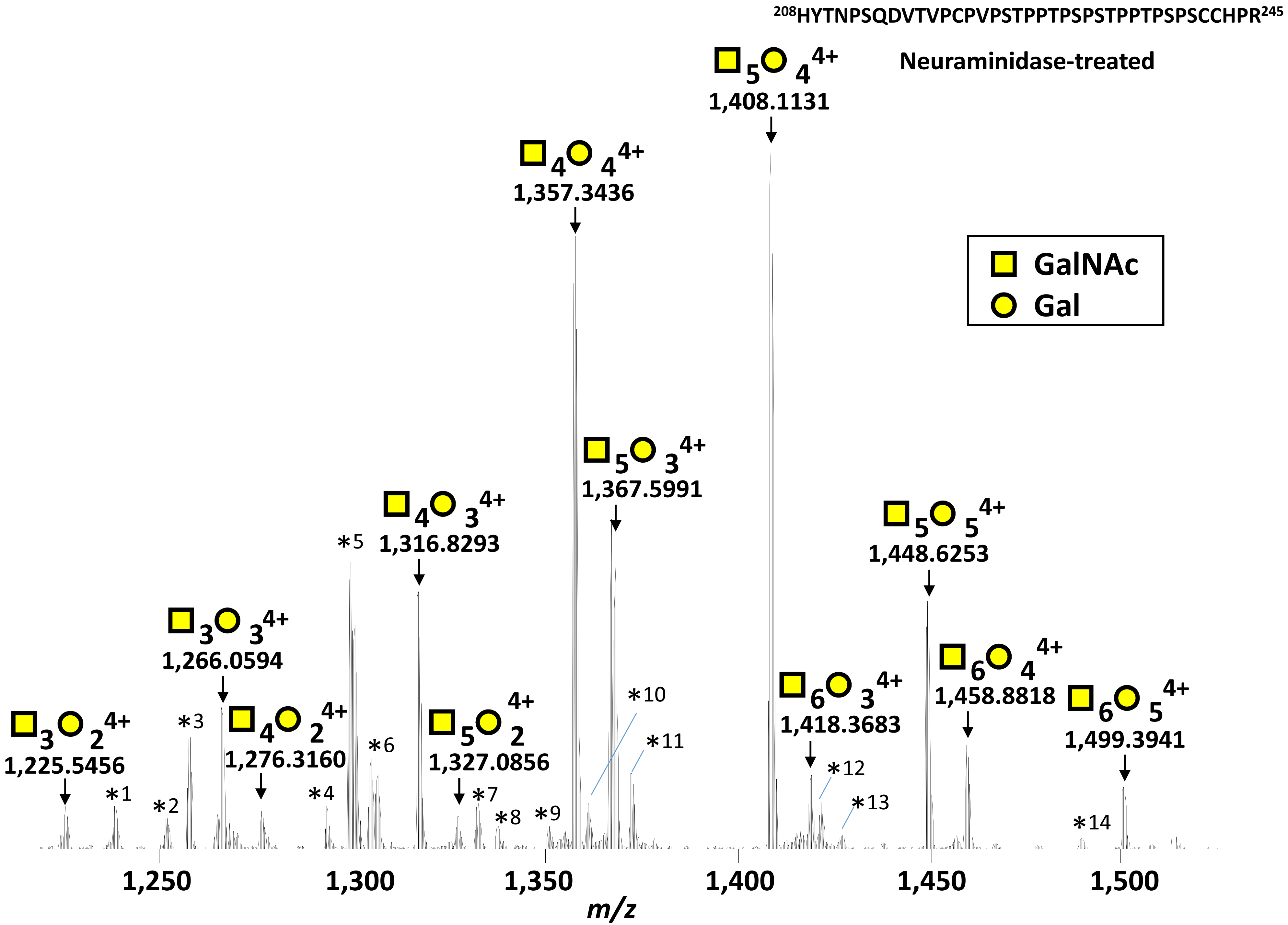

Supplementary Figure S4

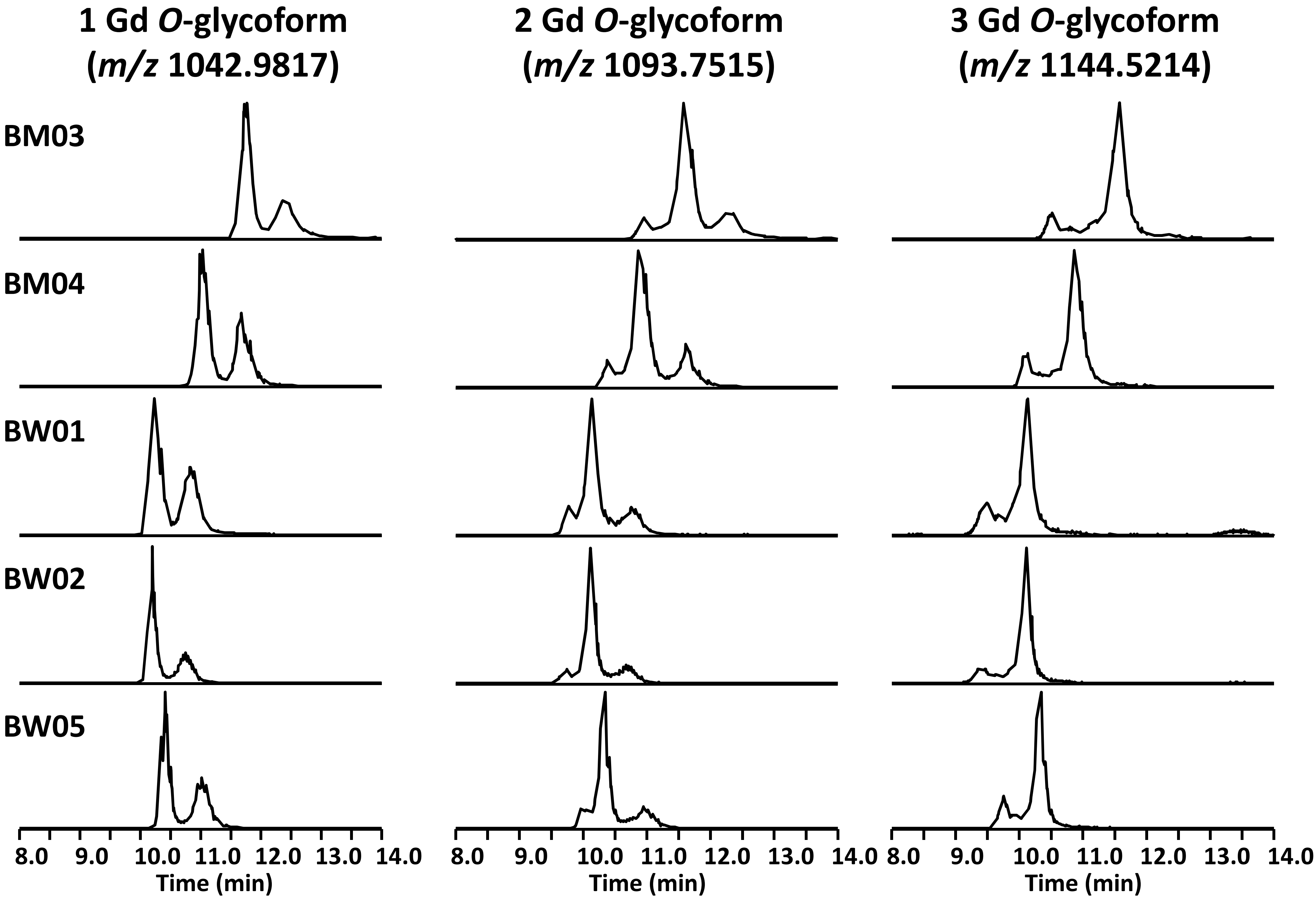

Supplementary Figure S5

Number of GalNAc_Gal/HR	*O*-glycanase from S. *pneumoniae*		*O*-glycanase from E. *faecalis*	
	Intensity	RA (%)	Intensity	RA (%)
0_0	1.4.E+09	13.68	2.8.E+09	45.95
1_0	1.6.E+09	16.28	2.6.E+09	41.93
1_1	2.0.E+09	20.56	7.0.E+06	0.11
2_0	4.9.E+08	4.93	6.3.E+08	10.23
2_1	1.3.E+09	13.32	2.6.E+06	0.04
2_2	9.7.E+08	9.75	1.7.E+07	0.27
3_0	1.1.E+08	1.10	8.7.E+07	1.40
3_1	3.1.E+08	3.10	N.D.	
3_2	7.4.E+08	7.48	N.D.	
3_3	3.8.E+08	3.83	N.D.	
4_0	6.6.E+06	0.07	3.7.E+06	0.06
4_1	5.3.E+07	0.53	N.D.	
4_2	1.4.E+08	1.45	N.D.	
4_3	2.4.E+08	2.45	N.D.	
4_4	5.7.E+07	0.57	N.D.	
5_2	1.8.E+07	0.18	N.D.	
5_3	3.4.E+07	0.34	N.D.	
5_4	2.8.E+07	0.28	N.D.	
5_5	9.7.E+06	0.10	N.D.	
Total	9.9.E+09	100	6.2.E+09	100

Supplementary Table S1. Comparison of RA of each *O*-glycopeptide after sequential deglycosylation using *O*-glycanase from S. *pneumoniae* and *O*-glycanae form E. *faecalis*.

Number of GalNAc_Gal/HR	RA, determined by the Analyzer (%) *n* =1	RA, determined manually (%) *n* =1
0_0	36.34	33.18
1_0	43.63	43.9
2_0	15.43	17.23
3_0	4.29	5.27
4_0	0.23	0.3
5_0	0.03	0.04
6_0	n.d.	n.d.

Supplementary Table S2. Relative abundance (RA,%) of each *O* -glycopeptide after sequential deglycosylation, determined by an automated program (Glycan Analyzer) and manually. n.d., not detected.

Product ion	Number of GalNAc	Theoretical mass (*m/z*)	Detected ion (*m/z*)	Mass error (ppm)	RT (min)
$(c\,6)^{+1}$	0	596.3402	596.3391	-1.84	5.49-13.87
$(c\,6)^{+1}$	1	799.4196	–	–	–
$(c\,8)^{+1}$	0	794.4407	794.4392	-1.89	5.47-13.76
$(c\,8)^{+1}$	1	997.5201	997.5195	-0.60	5.63-5.77
$(c\,10)^{+1}$	0	978.5255	978.5245	-1.02	5.49-10.99
$(c\,10)^{+1}$	1	1181.6049	–	–	–
$(c\,11)^{+1}$	0	1065.5575	1065.5541	-3.19	5.49-13.36
$(c\,11)^{+1}$	1	1268.6369	1268.6330, 1268.6302	-3.07, -5.28	5.63-5.80, 6.19-8.01
$(c\,14)^{+1}$	0	1360.7107	1360.7067	-2.94	5.74-6.17
$(c\,14)^{+1}$	1	1563.7901	1563.7744, 1563.7816	10.04, -5.45	6.52, 6.89
$(c\,16)^{+1}$	0	1558.8111	–	–	–
$(c\,16)^{+1}$	1	1761.8905	1761.8907	0.11	5.53-8.63

Product ion	Number of GalNAc	Theoretical mass (*m/z*)	Detected ion (*m/z*)	Mass error (ppm)	RT (min)
$(z\,18{+}1)^{+1}$	1	2036.9052	2036.907	0.88	5.47-11.74
$(z\,18{+}1)^{+1}$	0	1833.8258	–	–	–
$(z\,16{+}1)^{+1}$	1	1838.8048	1838.8084	1.96	5.55-10.60
$(z\,16{+}1)^{+1}$	0	1635.7254	1635.7296	2.57	5.66-5.76
$(z\,14{+}1)^{+1}$	1	1654.7200	1654.7206	0.06	5.55-9.06
$(z\,14{+}1)^{+1}$	0	1451.6406	1451.6523	8.06	6.58-7.34
$(z\,13{+}1)^{+1}$	1	1567.6880	1567.6856	-1.53	5.53-10.73
$(z\,13{+}1)^{+1}$	0	1364.6086	1364.6082, 1364.6030	-0.29, -4.10	5.63-5.82, 6.18-8.61
$(z\,10{+}1)^{+1}$	1	1272.5348	1272.5473	9.82	5.58-6.21
$(z\,10{+}1)^{+1}$	0	1069.4554	1069.4669, 1069.4613	10.75, 5.52	5.68, 6.18-9.82
$(z\,8{+}1)^{+1}$	1	1074.4343	–	–	–
$(z\,8{+}1)^{+1}$	0	871.3549	871.3589	4.59	5.51-10.73

Supplementary Table S3. The theoretical product ion mass (*m/z*) derived from the precursor ion corresponding to 1 Gd *O*-glycoform (*m/z* 877.7456). The mass value (*m/z*) of the detected fragment ion, mass error (ppm), and retention time (RT, min) are shown.

Product ion	Number of GalNAc	Theoretical mass (*m/z*)	Detected ion (*m/z*)	Mass error (ppm)	RT (min)
$(c\,6)^{+1}$	0	596.3402	596.3387	-2.52	5.66-9.97
$(c\,6)^{+1}$	1	799.4196	-	-	-
$(c\,6)^{+1}$	2	1002.4990	-	-	-
$(c\,8)^{+1}$	0	794.4407	794.4398	-1.13	5.98-10.08
$(c\,8)^{+1}$	1	997.5201	997.5172	-2.91	5.49-6.37
$(c\,8)^{+1}$	2	1200.5995	-	-	-
$(c\,10)^{+1}$	0	978.5255	978.5219	-3.68	6.30-6.95
$(c\,10)^{+1}$	1	1181.6049	1181.6024, 1181.5981, 1181.6005	-2.12, -5.75, -3.72	5.74-6.01, 6.48-6.73, 7.30-8.43
$(c\,10)^{+1}$	2	1384.6843	1384.6831	-0.87	6.14-6.32
$(c\,11)^{+1}$	0	1065.5575	1065.5548	-2.53	6.32-6.98
$(c\,11)^{+1}$	1	1268.6369	-	-	-
$(c\,11)^{+1}$	2	1471.7163	1471.7144, 1471.7213	-1.29, 3.40	6.06-6.37, 7.03-7.25
$(c\,14)^{+1}$	0	1360.7107	-	-	-
$(c\,14)^{+1}$	1	1563.7901	1563.7965, 1563.7777	4.09, -7.93	5.79-5.98, 6.34-6.60
$(c\,14)^{+1}$	2	1766.8695	1766.8520, 1766.8655	-9.90, -2.26	6.22-6.30, 7.06-7.72
$(c\,16)^{+1}$	0	1558.8111	-	-	-
$(c\,16)^{+1}$	1	1761.8905	-	-	-
$(c\,16)^{+1}$	2	1964.9699	1964.9663	-1.83	5.73-8.51

Product ion	Number of GalNAc	Theoretical mass (*m/z*)	Detected ion (*m/z*)	Mass error (ppm)	RT (min)
$(z\,18{+}1)^{+1}$	2	2239.9846	2239.9942	4.29	5.73-10.31
$(z\,18{+}1)^{+1}$	1	2036.9052	-	-	-
$(z\,18{+}1)^{+1}$	0	1833.8258	-	-	-
$(z\,16{+}1)^{+1}$	2	2041.8842	2041.8770	-3.53	6.29-7.94
$(z\,16{+}1)^{+1}$	1	1838.8048	1838.8095	2.56	5.49-6.33
$(z\,16{+}1)^{+1}$	0	1635.7254	-	-	-
$(z\,14{+}1)^{+1}$	2	1857.7994	1857.8079, 1857.7899	4.58, -6.19	6.37, 6.77
$(z\,14{+}1)^{+1}$	1	1654.72	1654.7173, 1654.7177, 1654.7342	-1.63, -1.39, 8.58	5.73-6.11, 6.40-6.78, 7.04-7.63
$(z\,14{+}1)^{+1}$	0	1451.6406	1451.6375	-2.14	6.19-6.29
$(z\,13{+}1)^{+1}$	2	1770.7674	1770.7855	10.22	6.47
$(z\,13{+}1)^{+1}$	1	1567.688	1567.6837, 1567.6824, 1567.6917	-2.74, -3.57, 2.36	5.51--6.19, 6.30-7.21, 7.36-8.71
$(z\,13{+}1)^{+1}$	0	1364.6086	1364.6060, 1364.6083, 1364.6084	-1.91, -0.22, -0.15	6.09-6.39, 6.93-7.62, 8.9-8.96
$(z\,10{+}1)^{+1}$	2	1475.6142	-	-	-
$(z\,10{+}1)^{+1}$	1	1272.5348	1272.5360, 1272.5298	0.94, -3.93	5.77-5.96, 6.28-6.75
$(z\,10{+}1)^{+1}$	0	1069.4554	1069.4532, 1069.4470	-2.06, -7.85	6.12-6.37, 6.80-8.45
$(z\,8{+}1)^{+1}$	2	1277.5137	-	-	-
$(z\,8{+}1)^{+1}$	1	1074.4343	-	-	-
$(z\,8{+}1)^{+1}$	0	871.3549	871.3535	-1.61	5.68-9.58

Supplementary Table S4. The theoretical product ion mass (*m/z*) derived from the precursor ion corresponding to 2 Gd *O*-glycoform (*m/z* 945.4388). The mass value (*m/z*) of the detected fragment ion, mass error (ppm), and retention time (RT, min) are shown.

Product ion	Number of GalNAc	Theoretical mass (*m/z*)	Detected ion (*m/z*)	Mass error (ppm)	RT (min)
$(c\,6)^{+1}$	0	596.3402	596.3385	-2.85	5.48-8.47
$(c\,6)^{+1}$	1	799.4196	–	–	
$(c\,6)^{+1}$	2	1002.4990	–	–	
$(c\,6)^{+1}$	3	1205.5784	–	–	–
$(c\,8)^{+1}$	0	794.4407	794.4400	-0.88	6.03-8.27
$(c\,8)^{+1}$	1	997.5201	997.517	-3.11	5.48-6.21
$(c\,8)^{+1}$	2	1200.5995	–	–	–
$(c\,8)^{+1}$	3	1403.6789	–	–	–
$(c\,10)^{+1}$	0	978.5255	978.5267	1.23	6.03-6.15
$(c\,10)^{+1}$	1	1181.6049	1181.6008	-3.47	6.31-6.97
$(c\,10)^{+1}$	2	1384.6843	1384.6707	-9.82	5.50-6.15
$(c\,10)^{+1}$	3	1587.7637	–	–	–
$(c\,11)^{+1}$	0	1065.5575	–	–	–
$(c\,11)^{+1}$	1	1268.6369	1268.6288	-6.38	6.34-6.83
$(c\,11)^{+1}$	2	1471.7163	1471.7061, 1471.7185	-6.93, 1.49	5.35-5.85, 6.25-6.40
$(c\,11)^{+1}$	3	1674.7957	1674.7922	-2.09	5.85-6.05
$(c\,14)^{+1}$	0	1360.7107	-	-	-
$(c\,14)^{+1}$	1	1563.7901	–	–	-
$(c\,14)^{+1}$	2	1766.8695	1766.8716, 1766.8491	1.19, -0.45	5.69, 6.43-6.67
$(c\,14)^{+1}$	3	1969.9489	1969.9453	-1.83	5.37-5.46
$(c\,16)^{+1}$	0	1558.8111	–	–	
$(c\,16)^{+1}$	1	1761.8905	–	–	
$(c\,16)^{+1}$	2	1964.9699	–	–	
$(c\,16)^{+1}$	3	2168.0493	2168.0454, 2168.0434	-1.80, -2.72	5.52-5.72,6.42-6.81

Product ion	Number of GalNAc	Theoretical mass (*m/z*)	Detected ion (*m/z*)	Mass error (ppm)	RT (min)
$(z\,18{+}1)^{+1}$	3	2443.0640	2443.0729	3.64	5.50-7.63
$(z\,18{+}1)^{+1}$	2	2239.9846	–	–	–
$(z\,18{+}1)^{+1}$	1	2036.9052	–	–	–
$(z\,18{+}1)^{+1}$	0	1833.8258	–	–	–
$(z\,16{+}1)^{+1}$	3	2244.9636	2244.9598	-1.69	6.24-6.81
$(z\,16{+}1)^{+1}$	2	2041.8842	2041.8824	-0.88	5.50-5.87
$(z\,16{+}1)^{+1}$	1	1838.8048	–	–	–
$(z\,16{+}1)^{+1}$	0	1635.7254	–	–	–
$(z\,14{+}1)^{+1}$	3	2060.8788	–	–	–
$(z\,14{+}1)^{+1}$	2	1857.7994	1857.8037	2.31	6.37-6.73
$(z\,14{+}1)^{+1}$	1	1654.7200	1654.7186	-0.85	5.50-6.17
$(z\,14{+}1)^{+1}$	0	1451.6406	1451.6491	5.86	5.97-6.01
$(z\,13{+}1)^{+1}$	3	1973.8468	–	–	–
$(z\,13{+}1)^{+1}$	2	1770.7674	1770.7806	7.45	6.41-6.60
$(z\,13{+}1)^{+1}$	1	1567.6880	1567.6830, 1567.6843	-3.19, -2.36	5.46-5.97, 6.13-6.37
$(z\,13{+}1)^{+1}$	0	1364.6086	1364.6054	-2.34	5.81-6.13
$(z\,10{+}1)^{+1}$	3	1678.6936	–	–	–
$(z\,10{+}1)^{+1}$	2	1475.6142	–	–	–
$(z\,10{+}1)^{+1}$	1	1272.5348	1272.5365, 1272.5433	1.34, 6.68	5.52-5.95, 6.29-6.75
$(z\,10{+}1)^{+1}$	0	1069.4554	1069.4556, 1069.4503	0.19, -4.77	5.89-6.29, 6.71-7.20
$(z\,8{+}1)^{+1}$	3	1480.5931	–	–	–
$(z\,8{+}1)^{+1}$	2	1277.5137	–	–	–
$(z\,8{+}1)^{+1}$	1	1074.4343	–	–	–
$(z\,8{+}1)^{+1}$	0	871.3549	871.3534	-1.72	5.44-7.24

Supplementary Table S5. The theoretical product ion mass (*m/z*) derived from the precursor ion corresponding to 3 Gd *O*-glycoform (*m/z* 1,013.13198). The mass value (*m/z*) of the detected fragment ion, mass error (ppm), and retention time (RT, min) are shown.

Analysis of *O*-glycoforms of the IgA1 hinge region by sequential deglycosylation

Supplementary Information

Yukako Ohyama[1], Hisateru Yamaguchi[2], Kazuki Nakajima[3], Tomohiro Mizuno[4], Yukihiro Fukamachi[5], Yasuto Yokoi[5], Naotake Tsuboi[1], Daijo Inaguma[1], Midori Hasegawa[1], Matthew B. Renfrow[6], Jan Novak[6], Yukio Yuzawa[1], Kazuo Takahashi[1,6,7*]

[1]Department of Nephrology, Fujita Health University School of Medicine, Toyoake, Japan

[2]Institute for Comprehensive Medical Science, Fujita Health University, Toyoake, Japan

[3]Center for Research Promotion and Support, Fujita Health University, Toyoake, Japan

[4]Analytical Pharmacology, Faculty of Pharmacy, Meijo University, Nagoya, Japan

[5]Mitsui Knowledge Industry, Tokyo, Japan

[6]Departments of Biochemistry and Molecular Genetics and Microbiology, University of Alabama at Birmingham, Birmingham, AL, USA

[7]Department of Biomedical Molecular Sciences, Fujita Health University School of Medicine, Toyoake, Japan

Supplementary Table and Figure Legends

Supplementary Figures

Supplementary Figure S1. Extracted ion chromatograms (XIC) of each product ion of the one-galactose–deficient (Gd) *O*-glycoform.

The XIC of product ions are shown within the tolerance of ±10 ppm. The attachment sites were identified based on a combination of *c* and *z* fragments. The *c* ions (c_6–c_{14}) without *N*-acetylgalactosamine (GalNAc) and c_{16} with one GalNAc residue were detected in the first section of XIC [retention time (RT), 5.5–6.2 min]. Further, *z* ions (z_{10}–z_{18}) with one GalNAc residue and z_8 without GalNAc were identified, as a mirror image of the *c* ions. This indicated that GalNAc was attached at T^{236}. In addition, the *c* ions (c_8, c_{11}, and c_{16}) with one GalNAc residue and *z* ions (z_8–z_{13} and z_{16}) without GalNAc were identified in a subsection of the first section (RT, 5.6–5.8 min). This indicated that in the 1 Gd *O*-glycoform detected in that section, Gd *O*-Glycan was attached mainly at T^{236} and partially at T^{228}. The *c* ions (c_6–c_8) without GalNAc and *c* ions (c_{11}–c_{16}) with one GalNAc residue were identified in the second section (RT, 6.2–13.9 min). The *c* ions (c_{10} and c_{11}) without GalNAc and *z* ions (z_{13} and z_{14}) with one GalNAc residue were detected (with low intensity) in this section. This indicated that in the latter section, the 1 Gd *O*-glycoform harbored a Gd *O*-Glycan mainly at S^{230}, and partially at T^{233} or S^{232} (Fig. 6a).

Supplementary Figure S2. The XIC of each product ion of the 2 Gd *O*-glycoform.

The XIC of product ions are shown within the tolerance of ±10 ppm. To identify the attachment sites of the 2 Gd *O*-glycoform, the presence or absence of the *c* ions with up to two GalNAc residues and *z* ions with up to two GalNAc residues were determined based on the theoretical product ion mass (Supplementary Table S3). To demonstrate the detection time of product ions, the XIC of each product ion was generated (Supplementary Fig. S2). Detection of c_6 without GalNAc, *c* ions (c_8, c_{10}, and c_{14}) with one GalNAc residue, and c_{16} with two GalNAc residues indicated that in the 2 Gd *O*-glycoform in the first section of XIC (RT, 5.5–6.1 min), the Gd *O*-glycans were attached at T^{228} and T^{236}. For the second section (RT, 6.1–6.3 min), detection of c_6 without GalNAc, c_8 with one GalNAc residue, and *c* ions (c_{10}–c_{16}) with two GalNAc residues indicated that Gd *O*-glycans were located at T^{228} and S^{230}. In the third section (RT, 6.3–6.8 min), *c* ions (c_6–c_{11}) without GalNAc, c_{14} with one GalNAc residue, and c_{16} with two GalNAc residues were identified. The *z* ions (z_{13}–z_{18}) with two GalNAc residues, z_{10} with one GalNAc residue, and z_8 without GalNAc were identified. In the same section, c_{10} with one GalNAc residue and *z* ions (z_{13} and z_{14}) with one GalNAc residue were also identified. This indicated that in peptides in this section, the Gd *O*-glycan attachment sites were at S^{230} or T^{233}, and T^{236}. In the fourth section (RT, 6.8–10.3 min), *c* ions (c_6–c_8) without GalNAc, c_{10} with one GalNAc residue, and *c* ions (c_{11}–c_{16}) with two GalNAc residues were detected. The *z* ions

(z_{16}–z_{18}) with two GalNAc residues, z_{14} with one GalNAc residue, and *z* ions (z_8–z_{10}) without GalNAc were also identified. This indicated the presence of Gd *O*-glycan attachment sites at S^{230} and T^{233}. In the same section, z_{13} without GalNAc was also detected, albeit as a minor species. This indicated that the 2 Gd *O*-glycoform in the fourth section contained minor species with the glycan attached at S^{230} and S^{232} instead of T^{233} (Fig. 6b).

Supplementary Figure S3. The XIC of each product ion of the 3 Gd *O*-glycoform.
The XIC of product ions are shown within the tolerance of ±10 ppm. To identify the attachment sites of the 3 Gd *O*-glycoform, the presence or absence of *c* ions with up to three GalNAc residues and *z* ions with up to three GalNAc residues were determined based on the theoretical product ion mass (Supplementary Table S3). To demonstrate the detection time of product ions, the XIC of each product ion was generated (Supplementary Fig. S3). In the first section of XIC (RT, 5.4–5.8 min), c_6 without GalNAc, c_8 with one GalNAc residue, *c* ions (c_{10}–c_{14}) with two GalNAc residues, and c_{16} with three GalNAcs residues were identified. Further, z_{18} with three GalNAc residues, z_{16} with two GalNAc residues, *z* ions (z_{10}–z_{14}) with one GalNAc residue, and z_8 without GalNAc were detected. This indicated that in the 3 Gd *O*-glycoform in the first section, the attachment sites were at T^{228}, S^{230}, and T^{236}. In the second section (RT, 5.8–6.1 min), c_6 without GalNAc, c_8 with one GalNAc residue, c_{10} with two GalNAc residues, and c_{11} with three GalNAc residues were detected. Similar, z_{18} with three GalNAc residues, z_{14} with one GalNAc residue, and *z* ions (z_8–z_{13}) without GalNAc were identified. This indicated that the Gd *O*-glycan was located at T^{228}, S^{230}, and S^{232}. In the third section (RT, 6.1–6.7 min), *c* ions (c_6–c_8) without GalNAc, c_{10} with one GalNAc residue, c_{11} with one or two GalNAc residues, c_{14} with two GalNAc residues, and c_{16} with three GalNAc residues were detected. Further, *z* ions (z_{16}–z_{18}) with three GalNAc residues, z_{14} with two GalNAc residues, z_{13} with two or one GalNAc residue, z_{10} with one GalNAc residue, and z_8 without GalNAc were detected. This indicted that the Gd *O*-glycan was attached at S^{230}, T^{236}, and T^{233} or S^{232}. In the fourth section (RT, 6.7–8.5 min), detection of *c* ions (c_6–c_8) without GalNAc and *z* ions (z_8–z_{10}) without GalNAc indicated that S^{230}, S^{232} and T^{233} were glycosylated (Fig. 6c).

Supplementary Figure S4. Representative mass spectrum of desialylated tryptic fragment of IgA1 HR *O*-glycoforms. The monoisotopic *m/z* value of HR *O*-glycopeptide ions, the number of sugar moieties assigned and their charge numbers are shown above the individual peaks. Twelve mass peaks of HR with *O*-glycans were detected, representing molecules with three to six *O*-glycan chains with up to three Gd *O*-glycans. *Marks unassigned peaks that are triply charged ions were not (glyco)peptides derived from IgA1 HR. The information of monoisotopic *m/z* value of unassigned peaks are provided below.

*[1] 1238.5827, *[2]1251.8635, *[3]1257.5893, *[4]1293.2321, *[5]1299.2357, *[6]1304.5669, *[7]1332.2640,

*[8]1337.5954, *[9]1350.9012, *[10]1360.9246, *[11]1372.2600, *[12]1420.9452, *[13]1426.6148, *[14]1488.6379

Supplementary Figure S5. The extracted ion chromatogram (XIC) of 1-3 Gd *O*-glycans with tryptic digested HR (H^{208}-R^{245}) detected from five individual samples named (BM03, BM04, BW00, BW01, and BW02). The monoisotopic *m/z* values of 1-3 Gd *O*-glycoforms are 1042.9817, 1093.7515, and 1144.5214 respectively. We note the minor differences in eluting times that were dependent on the date of sample analysis, specifically on the conditions of LC column. Relative abundance of each isomeric glycoform of individual samples are shown in dataset 3.

Supplementary Tables (Supplementary_data.xls)

Supplementary Table S1. Comparison of RA of each *O*-glycopeptide of IgA myeloma protein Mce1 after sequential deglycosylation using *O*-glycanase from S. *pneumoniae* and *O*-glycanae form E. *faecalis*.

Supplementary Table S2. Relative abundance (RA,%) of each *O*-glycopeptide after sequential deglycosylation, determined by an automated program (Glycan Analyzer) and manually. n.d., not detected.

Supplementary Table S3. The theoretical product ion mass (*m/z*) derived from the precursor ion corresponding to 1 Gd *O*-glycoform (*m/z* 877.7456). The mass value (*m/z*) of the detected fragment ion, mass error (ppm), and retention time (RT, min) are shown.

Supplementary Table S4. The theoretical product ion mass (*m/z*) derived from the precursor ion corresponding to 2 Gd *O*-glycoform (*m/z* 945.4388). The mass value (*m/z*) of the detected fragment ion, mass error (ppm), and retention time (RT, min) are shown.

Supplementary Table S5. The theoretical product ion mass (*m/z*) derived from the precursor ion corresponding to 3 Gd *O*-glycoform (*m/z* 1,013.13198). The mass value (*m/z*) of the detected fragment ion, mass error (ppm), and retention time (RT, min) are shown.
